# Lactoferrin affects rhinovirus B-14 entry into H1-HeLa cells

**DOI:** 10.1007/s00705-021-04993-4

**Published:** 2021-02-19

**Authors:** Caio Bidueira Denani, Antonio Real-Hohn, Carlos Alberto Marques de Carvalho, Andre Marco de Oliveira Gomes, Rafael Braga Gonçalves

**Affiliations:** 1 Instituto de Tecnologia em Imunobiológicos, Fundação Oswaldo Cruz, Rio de Janeiro, RJ Brazil; 2 Center for Medical Biochemistry, Max Perutz Laboratories, Medical University of Vienna, Vienna Biocenter, Vienna, Austria; 3 Departamento de Patologia, Centro de Ciências Biológicas e da Saúde, Universidade do Estado do Pará, Belém, PA Brazil; 4 Centro Universitário Metropolitano da Amazônia, Instituto Euro-Americano de Educação, Ciência e Tecnologia, Belém, PA Brazil; 5 Programa de Biologia Estrutural, Instituto de Bioquímica Médica Leopoldo de Meis, Centro de Ciências da Saúde, Universidade Federal do Rio de Janeiro, Rio de Janeiro, RJ Brazil; 6 Instituto Nacional de Ciência e Tecnologia de Biologia Estrutural e Bioimagem, Rio de Janeiro, RJ Brazil; 7 Departamento de Bioquímica, Instituto Biomédico, Universidade Federal do Estado do Rio de Janeiro, Rio de Janeiro, RJ Brazil

## Abstract

Lactoferrin is part of the innate immune system, with antiviral activity against numerous DNA and RNA viruses. Rhinoviruses, the leading cause of the common cold, are associated with exacerbation of respiratory illnesses such as asthma. Here, we explored the effect of bovine lactoferrin (BLf) on RV-B14 infectivity. Using different assays, we show that the effect of BLf is strongest during adhesion of the virus to the cell and entry. Tracking the internalisation of BLf and virus revealed a degree of colocalisation, although their interaction was only confirmed *in vitro* using empty viral particles, indicating a possible additional influence of BLf on other infection steps.

**Supplementary Information:**

The online version contains supplementary material available at 10.1007/s00705-021-04993-4.

## Introduction

The antiviral effect of the 80-kDa iron-binding glycoprotein lactoferrin (Lf) has been investigated for decades [1]. More recently, the effect of bovine Lf (BLf) on infection by different enteroviruses was also accessed. *In vitro* studies demonstrated that the presence of BLf during the viral adsorption phase suppresses infection by poliovirus strains Mahoney [2] and Sabin type I [3], coxsackievirus A16 [4], enterovirus A71 [5], and echovirus 6 [6] by restricting host cell entry. BLf sterically impedes receptor binding and/or competitively blocks interaction with sulfated glycosaminoglycans (GAGs), which might act as coreceptors or 'concentrating receptors' prior to the interaction with the specific virus receptor(s) that internalise the virus [7]. In a few instances, a post-adsorptive role of BLf has been reported, ranging from inhibition of echovirus 6 and enterovirus A71 uncoating [5, 8] and suppression of virus-induced apoptosis [9] to relatively minor unspecified intracellular effects on poliovirus Mahoney infection [2]. An *in vivo* protective role of BLf has been shown for mice challenged with a lethal dose of enterovirus A71 [5].

The above-mentioned effect of BLf has also been investigated in respiratory tract infections such as those caused by severe acute respiratory syndrome coronavirus and adenovirus type 2, showing impairment of viral infection *in vitro* [10, 11]. However, rhinoviruses (RV), which are important seasonal respiratory pathogens and a primary cause of the common cold [12], with more than 170 types, were nearly overlooked, even after the observation that some types can exploit GAGs as cellular (co)receptors [13].

RVs belong to the genus *Enterovirus*, and the species *Rhinovirus A* and *Rhinovirus B* can be divided, according to their cellular receptor, into a major group and a minor group, using intercellular adhesion molecule 1 (ICAM-1) and low-density-lipoprotein receptor (LDLR), respectively. Members of the more recently established species *Rhinovirus C* use cadherin-related protein 3 as a receptor. BLf has been proposed to interfere with heparan sulfate and LDLR during the first steps of Japanese encephalitis virus infection, with a negative effect on infection [14]. The critical role of sulfated GAGs was confirmed by treatment of host cells with sodium chlorate prior to infection [15]. However, no interaction with viruses using ICAM-1 as a receptor has been described so far.

Following entry by receptor-mediated endocytosis along diverse pathways [16], the native RV particle (N; VP1-VP4 + RNA) is first converted to a subviral A particle (VP1-VP3 + RNA) and then to a subviral B particle (VP1-VP3). Depending on the RV receptor group, the N-to-A particle conversion is triggered by host-cell receptor binding and facilitated by the low endosomal pH (major group), or exclusively by the acidic environment of the endosome (minor group). The latter was evaluated by using inhibitors of the vacuolar H^+^-ATPase (V-ATPase) or ammonium chloride, which consistently decreased uncoating of various RVs [17].

In this work, we evaluated the antiviral effect of BLf on different stages of infection by RV-B14 (an ICAM-1-binding, major-group virus) in addition to investigating the cellular distribution of BLf over time, concomitant with virus internalisation.

## Material and methods

### Cell culture and virus

H1-HeLa cells were cultured as monolayers in DMEM (Sigma-Aldrich, USA) supplemented with 10% foetal bovine serum (FBS, Thermo Fisher Scientific, USA) and 50 µg of gentamicin (Sigma-Aldrich, USA) per ml in a 5% CO_2_ humid atmosphere at 37 °C. For experiments with the virus, the incubator was set to 34 °C. The RV-B14 isolate used in this work was propagated and purified as described [18]. The purified RV-B14 used in the particle stability thermal release assay (PaSTRy) was donated by Dieter Blaas (Max Perutz Laboratories, Medical University of Vienna, Vienna Biocenter, Austria).

### Bovine lactoferrin (BLf)

Native BLf (30% iron saturation) containing capsules were purchased from Life Extension (USA). The capsules were dissolved in phosphate-buffered saline (PBS, pH 7.4) at 25 °C, and insoluble excipient material was removed by centrifugation at 5,000 rpm for 10 min, followed by the transfer of the supernatant to a fresh tube. The last two steps were repeated five times. The final supernatant was passed through a 0.2-µm filter, its concentration adjusted to 100 mg ml^−1^ with PBS, and aliquots were stored at 4 °C. BLf was labelled with FITC following a previously described protocol [19]. The native BLf used in PaSTRy and the lactoferrin binding assay was obtained from Art'Gerecht (Frankfurt, Germany). A freshly prepared 4 mg ml^−1^ solution in PBS was used for each assay.

### Cell viability assay

Cells cultivated in 24-well plates (TPP, Switzerland) to 90% confluence were incubated with increasing concentrations of BLf (0, 0.25, 0.5, 1.0, 2.0, and 3.0 mg ml^−1^) in infection medium (IM; high-glucose DMEM supplemented with 2% FBS, 30 mM MgCl_2_ and 50 µg of gentamicin per ml) for 5 days at 34 °C. On day 5, cells were stained for 1 h with crystal violet solution (0.2% [w/v] crystal violet, 150 mM NaCl, and 1% [v/v] formaldehyde). Subsequently, wells were extensively washed with water, and plates were scanned using an Odyssey CLx Imaging System (LI-COR, USA). As an additional test to evaluate cell viability, we measured the ATP content of cells grown to 90% confluency in 96-well plates and incubated them as described above in the presence of CellTiter-Glo (Promega Corporation, USA) according to the manufacturer's protocol. For each condition, the detection threshold was determined by addition of Triton X-100 directly to the wells (final concentration, 1%), followed by incubation for 2 hours at 34 °C. Both experiments were performed in three independent biological replicates, and the results were plotted and analysed using Prism 6 software (GraphPad, USA), showing the mean and standard deviation (Supplementary Fig. 1).

### Plaque reduction assay

The concentration of BLf used in the plaque reduction assay (1 mg ml^−1^) was selected based on the concentration used in the aforementioned work on BLf and enteroviruses, which ranged from 3 µg ml^−1^ to 3 mg ml^−1^. The approximate BLf concentration was employed in the H1-HeLa cell viability assay (Supplementary Fig. 1).

The plaque reduction assay was performed as described previously [20]. For the pre-adsorption assay, cells were incubated with 1 mg ml^−1^ BLf in IM for 60 min at 34 °C, washed with PBS, and incubated for 15 min at 4 °C with 50 plaque-forming units (PFU) of RV-B14 in IM per well to allow binding to the cells. The cells were kept at 34 °C for 60 min to trigger virus internalisation, washed, and covered with semisolid medium (0.8% carboxymethylcellulose in IM). For the adsorption assay, cells were incubated with RV-B14 plus 1 mg ml^−1^ BLf in IM for 15 min at 4 °C, followed by 60 min at 34 °C in the continuous presence of BLf, washed with PBS, and covered with semisolid medium. For the post-adsorption assay, cells were incubated with RV-B14 diluted in IM for 15 min at 4 °C and kept at 34 °C for 60 min. The medium was replaced by 1 mg ml^−1^ BLf in IM, and the cells were incubated at 34 °C for an additional 60 min, washed with PBS, and covered with semisolid medium. For testing the effect on all steps combined, cells were incubated with 1 mg ml^−1^ BLf in IM for 60 min, washed with PBS, incubated with RV-B14 in IM plus 1 mg ml^−1^ BLf for 15 min at 4 °C, and kept at 34 °C for another 60 min. Cells were washed with PBS, incubated with 1 mg ml^−1^ BLf at 34 °C for 60 min, washed again with PBS and covered with semisolid medium, after which the cells were incubated for 5 days at 34 °C and stained with 1% crystal violet solution. Plaque reduction due to BLf treatment was compared to the control condition without BLf but otherwise conducted identically, and the number of plaques was taken to represent 100% infection. To reach about 50 PFU of RV-B14 per well, we added 72 TCID_50_ by equating 1 TCID_50_ to 0.69 PFU [21]. Three independent biological replicates were performed for each condition. Mean values with error bars equivalent to ± 1 standard error (SE) are shown in the bar chart. Differences in the mean were evaluated by ANOVA with Sidak's multiple comparisons using Prism 6 software and were considered statistically significant for *p*-values ≤ 0.05.

### Direct evaluation of the effect of BLf on RV-B14 entry

The assay was performed as described previously [18]. In brief, cells (80% confluent) were incubated with RV-B14 (MOI = 100) in the presence or absence of 1 mg ml^−1^ BLf in IM for 15 min at 4 °C, followed by 30 min at 34 °C. Cells were washed with cold PBS, fixed with 3.8% formaldehyde in PBS, washed with PBS, and permeabilised in PBS with 0.1% Triton X-100. The fixed cells were again washed with PBS and processed at room temperature as follows: The samples were incubated with blocking buffer (5% BSA and 0.05% Tween-20 in PBS) for 2 h, followed by immunolabeling with rabbit anti-RV-B14 serum in blocking buffer (1:1000 dilution) for 1 h. After washing with PBS, cells were incubated with IRDye 680RD donkey anti-rabbit secondary antibody (LI-COR, USA) diluted in blocking buffer (1:2000 dilution) for 1 h. Nuclei were stained with 0.1% DAPI (Thermo Fisher Scientific, USA) in PBS for 10 min. Cells were then visualised under an LSM 510 Meta confocal fluorescence microscope (Zeiss, Germany). Nuclear staining and the bright-field channel were used to localise the cells. RV-B14 foci were counted manually for every cell present in 10 different fields in three separate preparations and plotted, and the statistical significance of differences in the mean of treated and untreated cells was analysed using Student *t*-test in Prism 6 software.

### Kinetics of BLf entry into cells and its dependence on sulfated GAGs

When the cells reached 60% confluence, the medium was replaced by DMEM enriched with 2% FBS plus 50 mM sodium chlorate (+NaClO_3_) or without sodium chlorate (-NaClO_3_), and the cells were incubated at 37 °C for 24 h. They were then washed and incubated with 1 mg ml^−1^ FITC-labelled BLf in DMEM containing 2% FBS +/- 50 mM sodium chlorate for 10 (T10), 30 (T30), or 60 (T60) min. Control cells (-BLf) were treated similarly to T60, but without the addition of FITC-labelled BLf. Cells were fixed as above and incubated with 0.1% Hoechst 33342 (Thermo Fisher Scientific, USA) in PBS for 10 min. Cells from 10 randomly picked fields (containing 5 to 10 cells per field) from two independent experiments for each condition were imaged using an LSM 510 Meta confocal fluorescence microscope. A representative field for each condition is shown.

### Tracking of RV-B14 and BLf during the early events of entry into cells

Cells were incubated with RV-B14 (MOI = 20) and 1 mg ml^−1^ FITC-labelled BLf in IM for 15 min at 4 °C and then at 34 °C for an additional 5 or 15 min. The cells were then washed with PBS, fixed, permeabilised, and incubated with blocking solution as described above. Next, the cells were immunolabeled with rabbit anti-RV-B14 primary antiserum (1:1000 dilution) for 1 h, incubated with IRDye 680RD donkey anti-rabbit secondary antibody (1:2000 dilution) for 1 h, and stained with 0.1% DAPI in PBS for 10 min. Cells were then visualised on an LSM 510 Meta fluorescence confocal microscope.

### PaSTRy

PaSTRy was performed as described [22] using a Bio-Rad CFX Connect Real-Time PCR instrument to detect the SYTO-82 (Thermo Fisher Scientific, USA) signal, indicating viral RNA accessibility. Purified RV-B14 (0.5 mg ml^−1^) was incubated with SYTO-82 (5 µM) in PBS +/- BLf (0.25, 0.5, and 1.0 mg ml^−1^) in 70 µl at the given final concentrations. Each measurement was carried out in triplicate (20 µl), and the RFU values were averaged to minimise measurement errors (technical replicates). The mean fluorescence (RFU) from two independent measurements was determined, and the corresponding first derivative was calculated and smoothed using GraphPad Prism 6.

### Lactoferrin binding assay

The assay was performed to determine (sub)viral particle binding to immobilised BLf in clear flat-bottomed high-binding 96-well plates (Corning, Sigma-Aldrich, USA). Plates were coated with 0.1 mg of BLf in PBS (pH 7.4) per well at 4 °C overnight. Positive control wells contained 10 µg of purified RV-B14 diluted in PBS per well instead of BLf and were incubated as above. Plates were washed with PBS to remove unbound BLf (and RV-B14) and blocked with 10% bovine serum albumin (BSA) for 2 h at 37 °C. Subsequently, plates were washed with PBS and (except for the positive control wells) incubated with 10 µg of purified RV-B14 (native) per well or a similar amount of subviral particles (A or B particles) in PBS for 1 h at 34 °C. The A particles were generated by incubation of purified RV-B14 (10 µg) with 100 µl of 100 mM sodium acetate buffer (pH 5.6) for 10 minutes at room temperature. The generated subviral particles were washed five times with PBS using Amicon Ultra Filters (Sigma-Aldrich, USA) for reneutralisation. The B particles were generated by heating purified RV-B14 (10 µg) to 56 °C for 10 min [22]. After washing with 1% BSA in PBS-T (PBS containing 0.05% Tween-20), plates were incubated with rabbit anti-RV-B14 serum [18] diluted (1:200) in PBS-T containing 1% BSA at 34 °C for 1 h. The plates were then washed and incubated with DyLight 800-conjugated anti-rabbit antibody (Thermo Fisher Scientific, USA) diluted 1:500 in PBS-T containing 1% BSA at 34 °C for 1 h. After washing, the plates were scanned using an Odyssey CLx Imaging System for fluorescence emission using the 800 nm channel. Each sample RFU was averaged from four wells to minimise measurement errors (technical replicates). A representative plate and a bar chart from three independent replicates displaying the respective mean values with error bars equivalent to ± 1 standard deviation (SD) are shown. Differences in the mean were evaluated by ANOVA with Sidak's multiple comparisons using Prism 6 software.

## Results

**Fig. 1:**
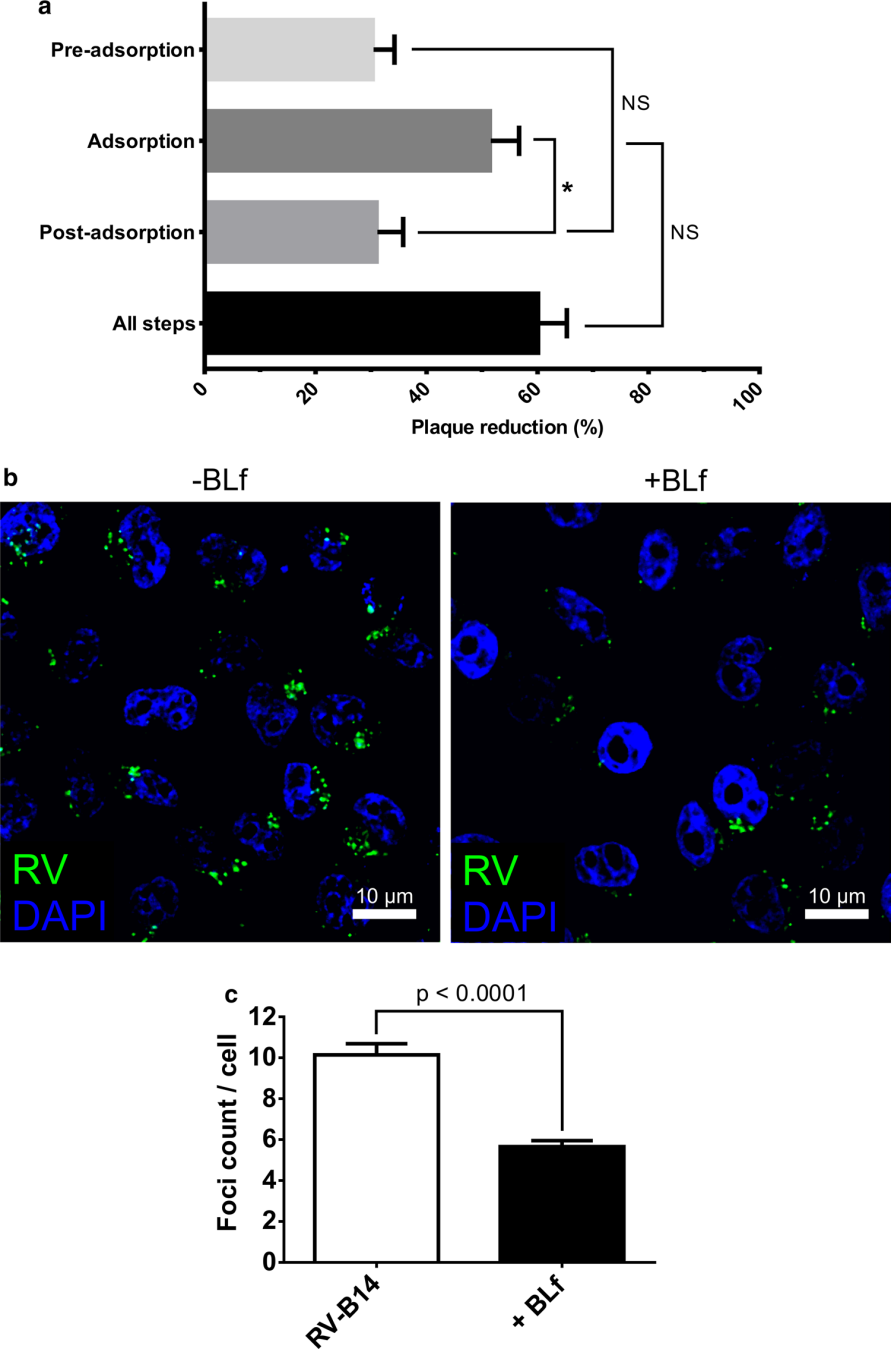
BLf affects RV-B14 infection primarily by interfering with cell entry. (a) Plaque reduction assay performed using H1-HeLa cells infected with RV-B14 and incubated with BLf at different steps of the infection: pre-adsorption, adsorption, post-adsorption, and all steps. (b) Identification of intracellular RV-B14 foci at 30 min postinfection in H1-HeLa cells incubated with 1 mg ml^−1^ BLf (+BLf) during virus adsorption. In the control (-BLf), BLf was not included. The panel shows representative confocal images, depicting RV-B14 particles (green) located intracellularly and detected using specific antibodies. The cell nucleus was labelled with DAPI (blue). (c) Quantification of virus foci per cell in the absence (empty bar) or presence (full bar) of BLf from 10 different fields of view in three separate preparations. *, *p* < 0.05; NS, not statistically significant

To test the effect of BLf on infection by the major-group virus RV-B14, we performed a plaque reduction assay by adding BLf at different steps of the infection cycle: pre-adsorption, adsorption, post-adsorption, and all steps (Fig. 1a). The strongest plaque reduction was observed when BLf was present during adsorption (~52%) and throughout (all steps; ~60%), with no statistically significant difference between the two treatments. However, both were significantly different from the more modest but consistent plaque reduction of ~30% in the pre-adsorption and post-adsorption schemes, while no statistically significant difference was found between those two latter schemes.

The substantial plaque reduction observed when BLf was present at the start of the infection, comparable to its continuous presence (the all-steps scheme), indicated that it predominantly interfered with an early step of the viral life cycle. This was further corroborated by confocal microscopy analysis of virus internalisation. RV-B14 at an MOI of 100 was attached to cells in the cold for 15 min in the presence or absence of BLf. Endocytosis was then triggered by warming the cells to 34 °C, and intracellular viral capsid proteins were detected 30 min post-entry by indirect immunofluorescence. This showed the presence of several bright foci within the infected cells (Fig. 1b), which most likely represented virus-loaded vesicles, as reported previously [18]. Quantification of foci in multiple cells revealed that the presence of BLf resulted in a decrease in RV-B14 entry by ~44% (Fig. 1c). Since virtually no surface-associated virus was detected, the result confirmed our hypothesis that BLf prominently acts during cell entry by significantly blocking virus attachment, but not its endocytosis once it is bound to the cells.

**Fig. 2:**
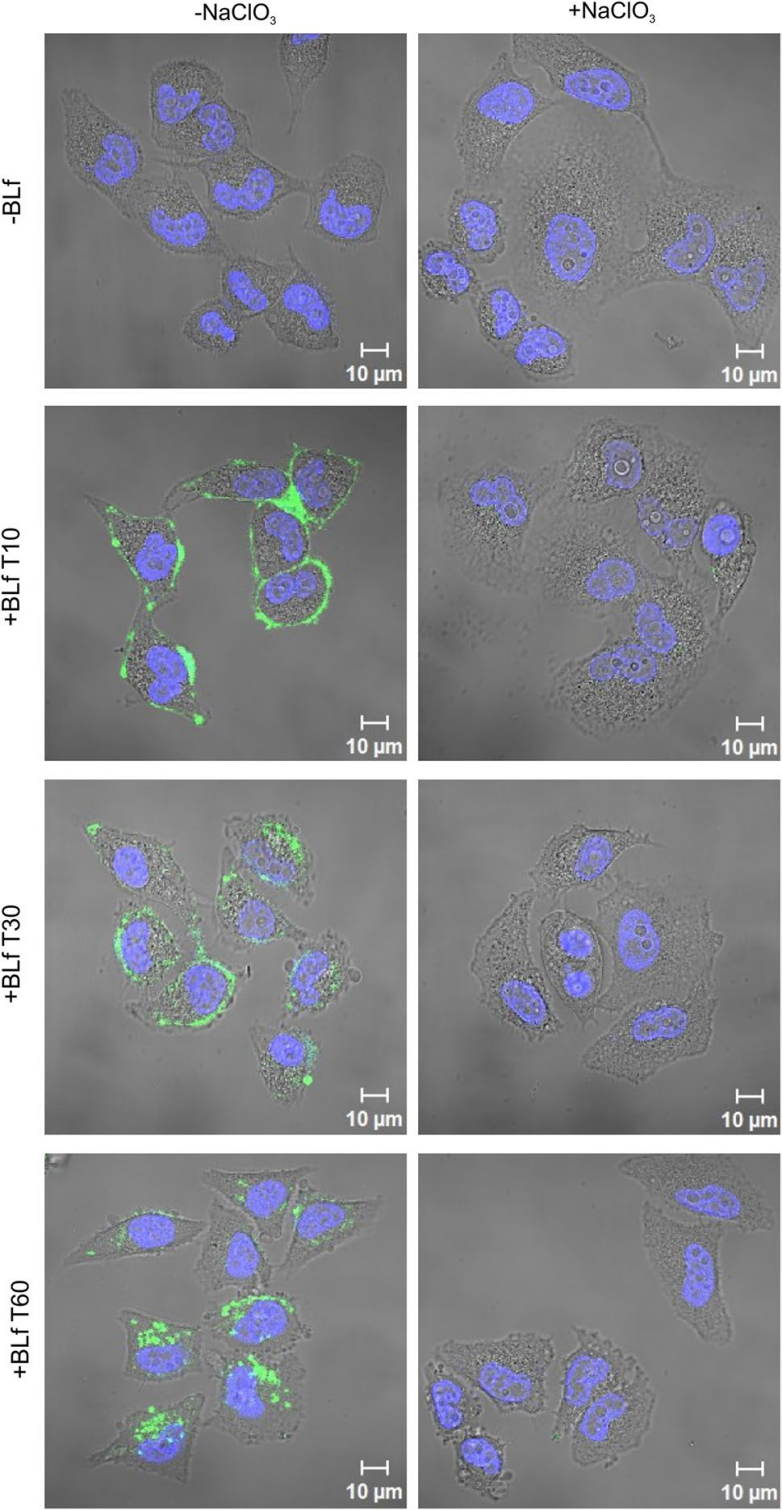
Binding and internalisation of BLf in H1-HeLa cells is dependent on the sulfation of GAGs. The role of GAG sulfation in the cellular binding of BLf was assessed by incubating the cells with conventional medium (-NaClO_3_) or medium supplemented with 50 mM sodium chlorate (+NaClO_3_) for 24 h before adding fresh medium (-BLf panels) or 1 mg ml^−1^ FITC-labelled BLf (+BLf panels) to the cells. The kinetics of BLf entry were determined by recording the fluorescent signal at 10, 30, and 60 min post-incubation. Representative fields depicting FITC-labelled BLf (green) and labelled nuclei (blue) are shown

Based on the above results, we next (indirectly) assessed whether BLf might perturb the interaction between the RV-B14 and its receptor, ICAM-1 [23]. By preventing the attachment to its main surface target, sulfated GAGs [19] (e.g., heparan sulfate presented by various proteoglycans), we evaluated whether some BLf remains bound to proteins expressed in HeLa cells, including ICAM-1. We achieved this by abolishing GAG sulfation by incubating the cells for 24 h with sodium chlorate. Then, cell monolayers were incubated with FITC-labelled BLf for 10, 30, and 60 min, and the fluorescent signal was visualised by confocal microscopy (Fig. 2). Untreated control cells demonstrated an even distribution of BLf over the cell membrane at 10 min, followed by uptake and accumulation in the perinuclear region (juxtaposed to or within the *cis*-Golgi region) at 30 min and 60 min post-binding, respectively. In stark contrast, no fluorescence was detectable after pretreatment with sodium chlorate, suggesting that BLf attachment to the cell membrane is critically dependent on the presence of sulfated GAGs. This result consequently ruled out a direct interaction between BLf and ICAM-1 on the HeLa cell membrane and thus competition with RV-B14 for binding to its entry receptor. The inhibitory effect of BLf on virus binding might therefore instead be due to steric hindrance by GAG-immobilised BLf decorating proteoglycans in the vicinity of ICAM-1, with residual binding/internalisation perhaps mediated by hypothetical coreceptors or 'concentrating receptors'.

**Fig. 3:**
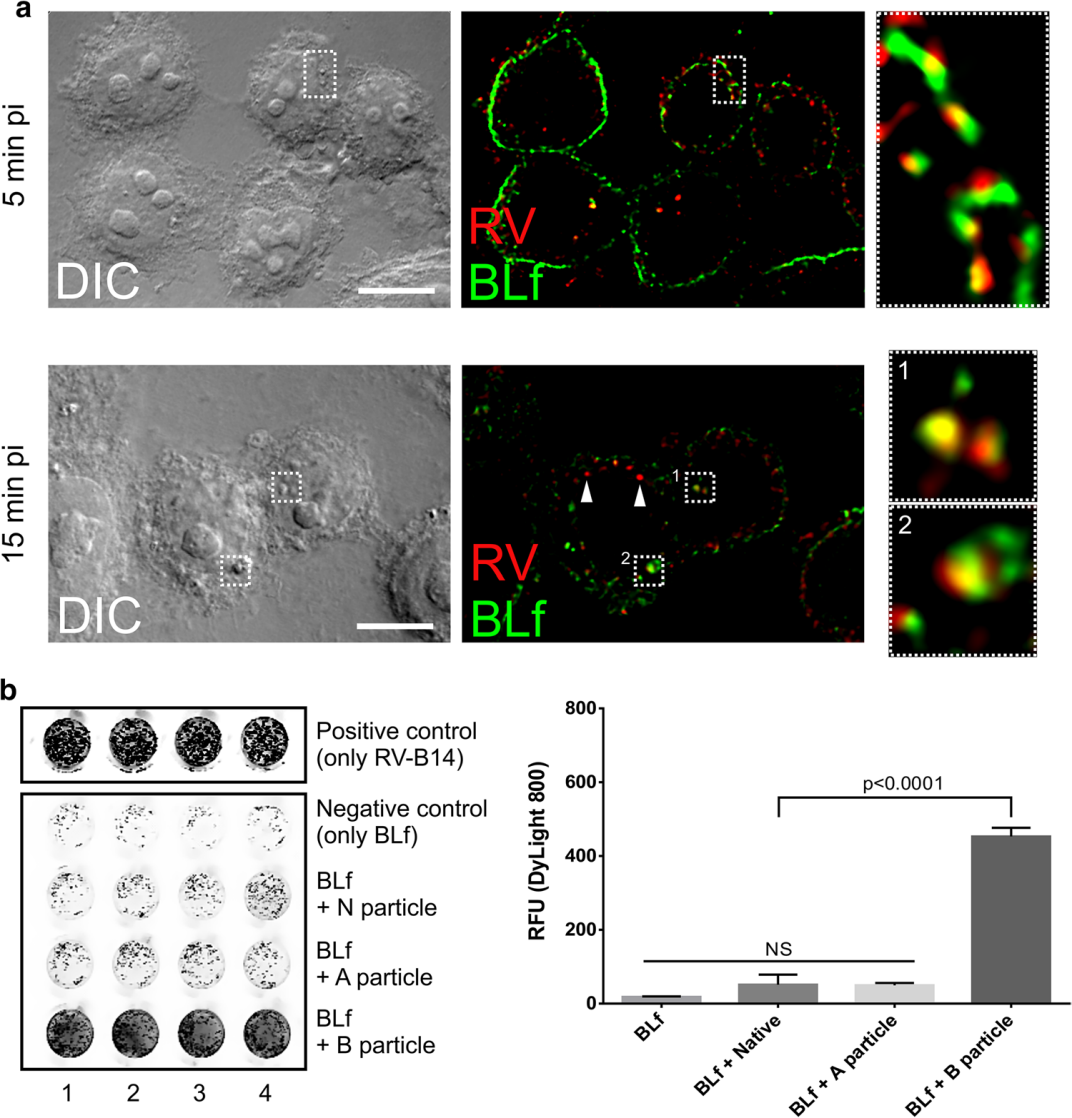
RV-B14 and BLf share similar entry routes but lack a direct interaction when probed *in vitro*. (a) H1-HeLa cells were incubated with RV-B14 and 1 mg ml^−1^ FITC-labelled BLf, and entry was imaged by confocal fluorescent microscopy at 5 (upper panels) and 15 (lower panels) min post-adsorption. The left panels are bright-field images, showing cell morphology and cell borders, and the middle panels and the insets in the right panels show the fluorescence signals of the RV-B14-specific antibodies (red) and FITC-labelled BLf (green) merged (individual fluorescent channels are shown in Supplementary Fig. 3). (b) BLf (0.1 mg) was immobilised to a 96-well plate overnight and subsequently incubated individually with native RV-B14 and A and B particles. (Sub)viral particle interaction was detected by immunofluorescence and quantified using ImageJ. Representative results (left) and mean and standard deviation quantification of three independent assays (right) are presented. As a positive control, one plate was incubated with RV-B14 overnight instead of BLf and processed similarly

As approximately 56% of the RV-B14 escaped being blocked by the presence of BLf during adsorption (Fig. 1b, c), we wondered whether uptake of BLf and simultaneous infection with RV-B14 would result in their colocalisation, thereby perhaps also interfering with subsequent stages, e.g., uncoating. This might then account for the slightly stronger effect of BLf in the plaque reduction assay (when present during adsorption or throughout) when compared to its effect on virus attachment (see above). To answer this question, we tracked RV-B14 and BLf during cell entry by fluorescence imaging of FITC-labelled BLf and RV-B14 (detected using a rabbit anti-RV-B14 serum followed by IRDye 680RD-labeled secondary antibody) at 5 and 15 min post-binding (Fig. 3a). We observed a non-uniform colocalisation in elongated cytoplasmic vesicles (resembling sorting endosomes – upper panel inset) at 5 min post-binding, which largely persisted at 15 min post-binding in mostly round vesicles (resembling endosomal carrier vesicles – lower panel insets). Some RV-B14-containing vesicles did not colocalise with BLf, which was more evident at 15 min post-binding (white arrowheads). Because of this considerable colocalisation of internalised RV-B14 with BLf, we also evaluated a direct action of BLf on RV-B14 uncoating intermediates, namely A and B particles. A direct interaction between BLf and N *in vitro* using an ELISA-based assay was not detected, in line with the PaSTRy experiment (Supplementary Fig. 2), nor was an interaction detected with A particles (Fig. 3b). Unexpectedly, however, we detected significant binding of B particles to BLf, although this is unlikely to be relevant for the uncoating of this virus.

## Discussion

Here, we demonstrate a distinct antiviral effect of BLf on infection of H1-HeLa cells by the major-group rhinovirus RV-B14. The interference with infectivity was highest when BLf was added to the cells together with the virus and maintained throughout the experiment (all steps), resulting in a ~60% drop in plaque formation. Insignificantly less reduction in plaque formation (~52%) was observed when BLf was present only during virus attachment and endocytic uptake (adsorption). The extent of this inhibition is comparable to previous results with poliovirus [2, 3].

Immunofluorescence analysis demonstrated a ~44% decrease in intracellular fluorescent foci, representing virus-containing vesicles [18], most likely resulting from inhibition of RV-B14 binding to the host cells. This effect consequently accounts for most of the plaque reduction under comparable conditions. Direct competition with ICAM-1 is unlikely to occur, as the elimination of cellular sulfation in HeLa cells via sodium chlorate treatment reduced the binding of fluorescently labelled BLf to the plasma membrane to undetectable levels, implicating sulfated GAGs as a main binding site. Additionally, no BLf internalisation was observed. Notably, HeLa cells express LDLR [24, 25], which is known to bind and internalise lactoferrin amongst several other ligands [14, 26]. Interestingly, chylomicron remnants were found to require prior binding to GAGs for subsequent transfer to LDLR, mediating their endocytosis into HepG2 cells. Heparinase treatment completely abolished their attachment to these cells [27]. We therefore speculate that BLf likewise requires initial binding to GAGs for subsequent shuttling to LDLR to explain the nearly complete loss of surface-associated fluorescent BLf from chlorate-treated HeLa cells. Our suggestion that RV-B14 could interact with the cell membrane regardless of the presence of ICAM-1 (e.g., via hypothetical coreceptors or 'concentrating receptors') is corroborated by the observation of binding of RV-B14 to COS-7 cells lacking ICAM-1 [28]. Other researchers demonstrated that competition between membrane-bound RV-B14 (on HeLa cells) and anti-ICAM-1 antibody removed only 80% of the virus, even when using 0.4 mg ml^−1^, a very high concentration of the antibody [23]. Both observations highlight the possibility of RV-B14 interacting with membranes without the assistance of the main receptor, which is in agreement with RV-B14-binding being competitively inhibited by sulfated GAG binding BLf or by steric hindrance of ICAM-1.

Cointernalisation of RV-B14 and FITC-labelled BLf demonstrated that, within the internalised fraction, some virus colocalised with BLf, presumably resulting from an occasionally shared entry pathway, as ICAM-1 binding RVs can enter HeLa cells via multiple pathways, as seen for RV-B14 and RV-A89 [29]. Direct association between virions and BLf, when located in the same vesicle, could potentially contribute to reduced infectivity. RV-B14 uncoating is complete at 15 min pi [18], notably, at a time when this colocalisation becomes rather pronounced. However, our *in vitro* assay did not indicate a direct interaction between RV-B14 N and A particles at neutral pH, similar to what has been observed for echovirus 6 [8]. However, in the same publication, Ammendolia et al. demonstrated that echovirus 6 could bind BLf exclusively at low pH (< 6), which is found in endosomal carrier vesicles. This supports our colocalisation data obtained at 15 min pi and might suggest an additional effect of BLf via interference with uncoating.

The BLf binding assay revealed that (empty) B particles could interact with immobilised BLf. To generate B particles, the RNA must be ejected, possibly through a hole opening via loss of one or more pentamers [30], giving immobilised BLf access to the external and internal faces of the free pentamers and increasing the likelihood of BLf interaction. This favours a putative interaction between BLf and viral structural proteins and suggests that virus translation and/or assembly could be additional targets of BLf. Furthermore, the above presents a reasonable explanation for the observed ~30% plaque reduction when BLf was present before or after virus attachment/entry, as seen for other enteroviruses [2, 5, 8], including RV-A2 (an LDLR-binding minor-group virus), where the addition of BLf (1 mg ml^−1^) 30 min prior to RV-A2 adsorption caused 10% plaque reduction [31]. The latter study, however, in contrast to our analysis with the major-group RV-B14, did not evaluate the effect of BLf during adsorption and post-adsorption.

Taken together, our results indicate that BLf acts mainly at the plasma membrane, presumably via steric hindrance by binding to sulfated GAGs, such as members of the syndecan family. The target(s) of its slight post-binding effect remain(s) to be identified.

## Supplementary Information

Below is the link to the electronic supplementary material.

Supplementary file1 (PDF 70 KB)

Supplementary file2 (PDF 89 KB)

Supplementary file3 (PDF 132 KB)

## Data Availability

All datasets presented in this study are included in the article/supplementary material.
