# Supplementary material for: Lactoferrin affects rhinovirus B-14 entry into H1-HeLa cells
Source: Arch Virol. 2021 Feb 19;166(4):1203–11. doi: 10.1007/s00705-021-04993-4 (PMC7894240; doi:10.1007/s00705-021-04993-4)
Supplement: Supplementary file 1 — Supplementary file1 (PDF 70 KB) [file 705_2021_4993_MOESM1_ESM.pdf]

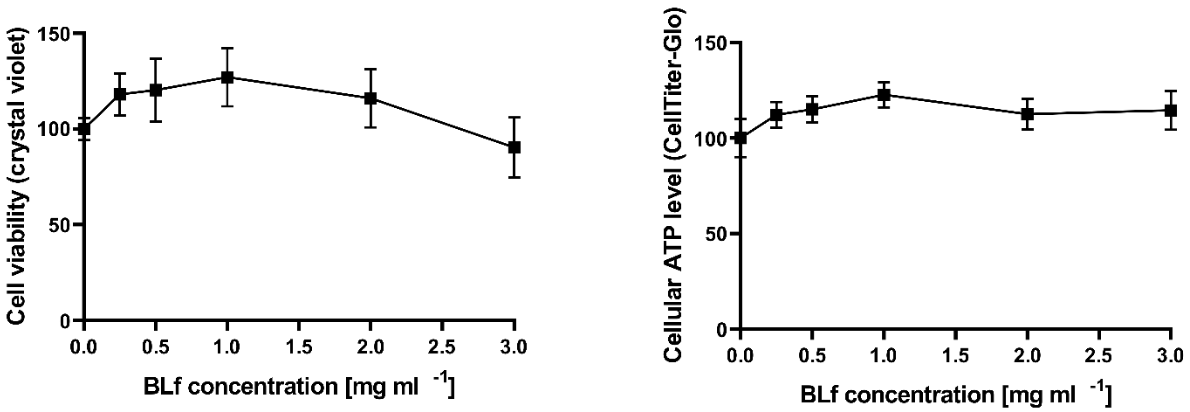

Supplementary Fig. 1. Cell viability assays using different concentrations of BLf for five days at 34 °C. HeLa cells were incubated with different BLf concentration (0 – 3 mg ml $^{-1}$) in infection medium (high glucose DMEM supplemented with 2% FBS, 30 mM $MgCl_2$ and 50 µg/mL gentamicin) for 5 days at 34 °C. On the day 5, cells were evaluated for viability using crystal violet solution (Left) and the level of cellular ATP using CellTiter-Glo reagent (Right). The data are presented as mean and standard deviation from 3 independent replicates (n = 3).
