# Supplementary material for: Lactoferrin affects rhinovirus B-14 entry into H1-HeLa cells
Source: Arch Virol. 2021 Feb 19;166(4):1203–11. doi: 10.1007/s00705-021-04993-4 (PMC7894240; doi:10.1007/s00705-021-04993-4)
Supplement: Supplementary file 2 — Supplementary file2 (PDF 89 KB) [file 705_2021_4993_MOESM2_ESM.pdf]

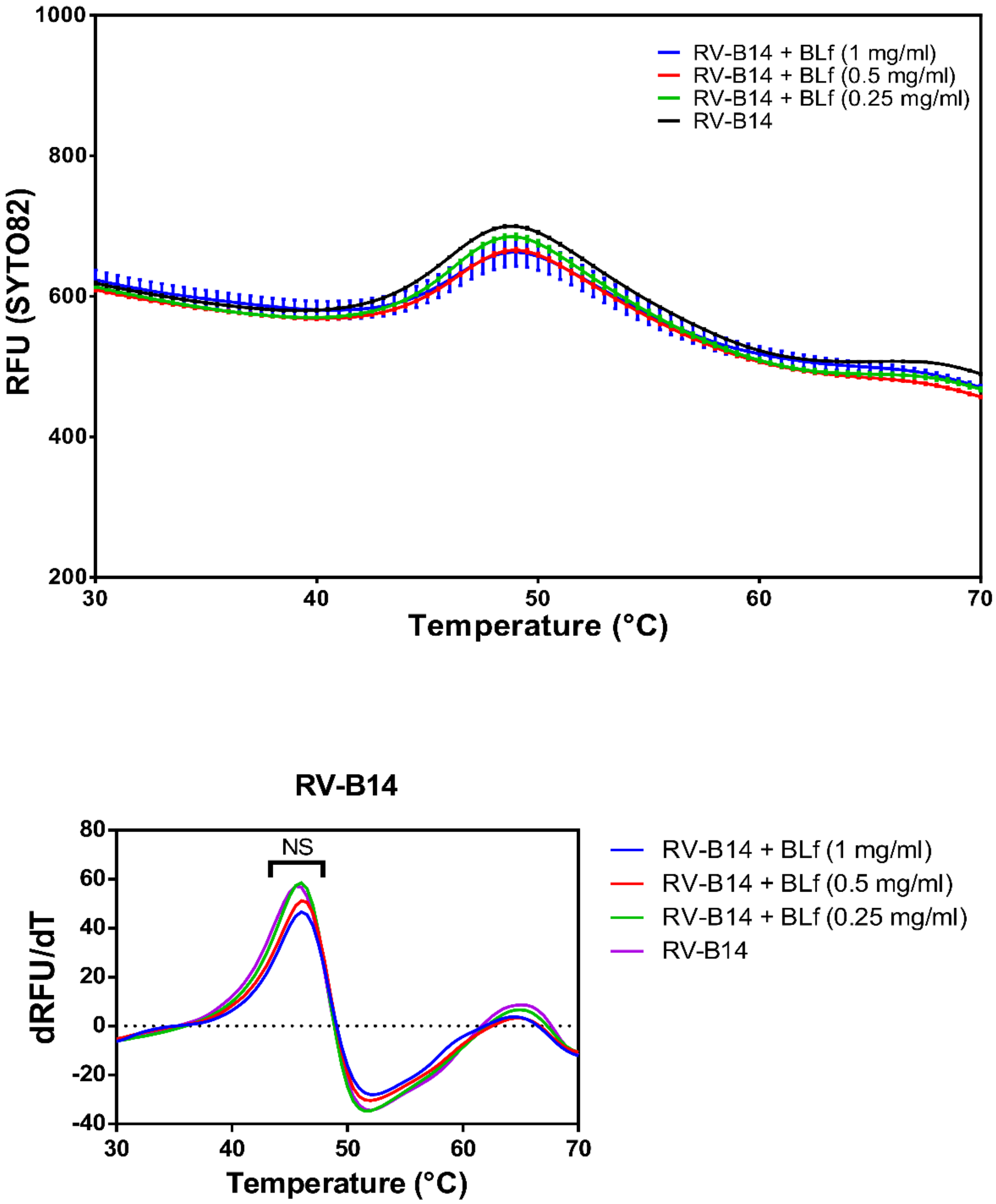

Supplementary Fig. 2. The PaSTRy of RV-B14 in the presence of BLf.

Fluorescence signal curves showing means with respective error bars equivalent to ± 1 standard error (upper panel) from 2 independent measurements, and corresponding first-order derivatives (Lower panel) are displayed.
