# Supplementary material for: Lactoferrin affects rhinovirus B-14 entry into H1-HeLa cells
Source: Arch Virol. 2021 Feb 19;166(4):1203–11. doi: 10.1007/s00705-021-04993-4 (PMC7894240; doi:10.1007/s00705-021-04993-4)
Supplement: Supplementary file 3 — Supplementary file3 (PDF 132 KB) [file 705_2021_4993_MOESM3_ESM.pdf]

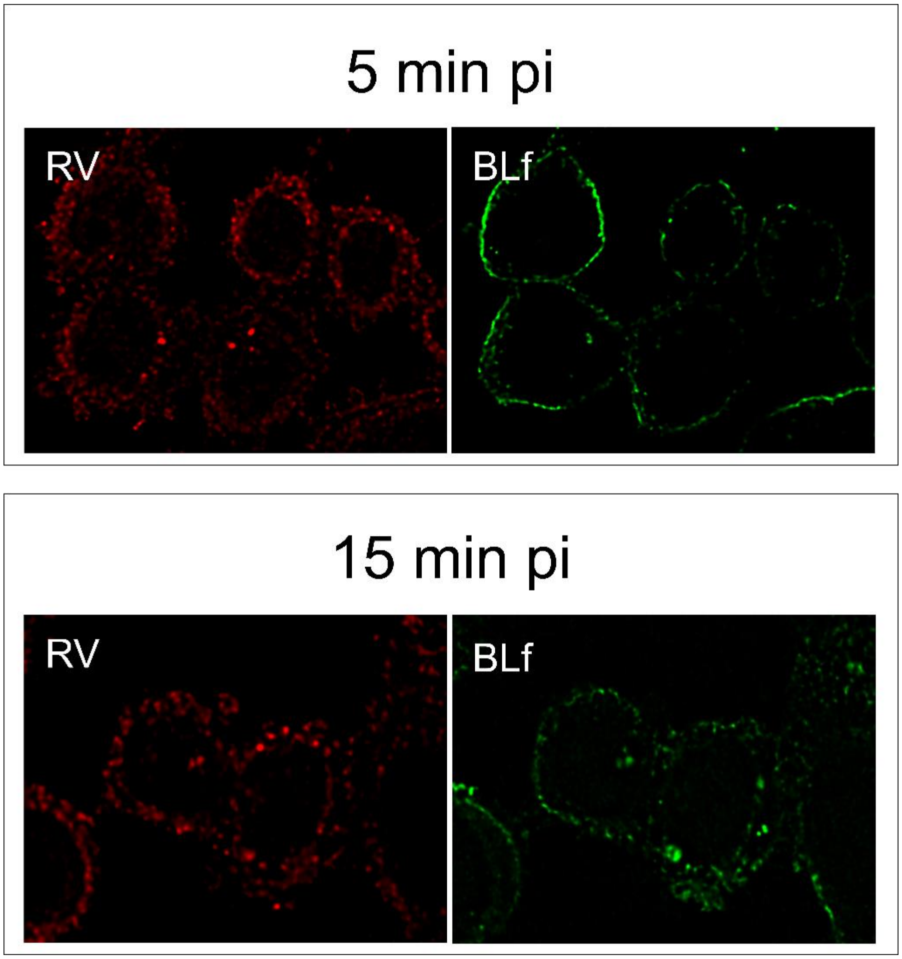

Supplementary Fig. 3. Split-channels of the Fig. 3a central panel. (Sub)cellular localization of fluorescence signals of anti-RV-B14 (rabbit serum followed by specific fluorophore conjugated secondary antibody – red) and FITC-labelled BLf (green) at 5 and 15 min pi.
